# The links between parental smoking and childhood obesity: data of the longitudinal study of Australian children

**DOI:** 10.1186/s12889-023-17399-5

**Published:** 2024-01-02

**Authors:** Preety Srivastava, Trong-Anh Trinh, Karen T. Hallam, Leila Karimi, Bruce Hollingsworth

**Affiliations:** 1 School of Economics, Finance and Marketing, RMIT University, Melbourne, Australia; 2 Centre for Health Economics, Monash University, Melbourne, Australia; 3 Division of Psychology, RMIT University, Melbourne, Australia; 4 Division of Health Research, Lancaster University, Lancaster, UK

**Keywords:** Child obesity, Parental Smoking, Nutrition, Taste preference, Endogeneity, C3, D1, I1

## Abstract

Childhood obesity is one of the most concerning public health issues globally and its implications on mortality and morbidity in adulthood are increasingly important. This study uses a unique dataset of Australian children aged 4–16 to examine the impact of parental smoking on childhood obesity. It confirms a significant link between parental smoking (stronger for mothers) and higher obesity risk in children, regardless of income, age, family size, or birth order. Importantly, we explore whether heightened preference for unhealthy foods can mediate the effect of parental smoking. Our findings suggest that increased consumption of unhealthy foods among children can be associated with parental smoking.

**Supplementary Information:**

The online version contains supplementary material available at 10.1186/s12889-023-17399-5.

## Introduction

Tobacco smoking is the leading cause of preventable disease, disability, and death worldwide. In June 2021, the World Health Organization estimated that over eight million people die prematurely due to tobacco use annually, which represents around 15% of global deaths [1]. According to the Australian Bureau of Statistics (ABS), in 2021-22, 10.1% or 1.9 million Australians aged 18 and older smoked tobacco daily [2].

Tobacco smoking has been identified as a potent predictor of childhood obesity, with one U.S. study showing parental smoking associated with a 40% increased risk of childhood obesity [3]. Children’s exposure to tobacco smoke has been associated with decreased brachial flow-mediated dilatation [4], increased risk of carotid atherosclerosis plaque [5], and impaired bone mineral density [6] in adulthood. It has also been linked with asthma and other respiratory conditions, middle ear infections, and conductive deafness [7–11]. Maternal tobacco use during the pregnancy period has likewise been linked with significant negative metabolic health outcomes in offspring, including increased body mass index (BMI), waist circumference [12], central adiposity and abdominal fat distribution in childhood [13–15] and adulthood [16]. This risk is likely associated with intra-uterine effects that are linked with lower infant birth weights and higher adiposity than infants from non-smoking mothers [17]. Genetic studies indicate that this may have some relationship with disruption in DNA methylation processes within infants exposed to tobacco smoke in utero [18]. Research with animal models further indicate altered hypothalamic functioning via modification of neuropeptide activity that relate to appetite in offspring [19, 20]. Beyond direct biological impacts of the uterine environment, a recent meta-analysis shows increased (but more modest) rates of child obesity when paternal smoking is present [21], indicating the possibility of both in utero and environmental impacts.

Australia has one of the highest rates of childhood obesity among high income nations, with the latest data indicating that approximately one in four Australian children are overweight or obese [22]. Obesity in children can lead to a range of metabolic and cardiovascular risks alongside some cancers, and increase the risk of gastrointestinal disease, which can persist into adulthood [12, 23]. Obesity can also have negative emotional and social impacts on children, such as low self-esteem and increased rates of being bullied and socially excluded with further adverse consequences on academic performance and long-term employment opportunities [24–27].

In relation to tobacco smoking, there are a number of theories explaining the links between parental smoking and childhood obesity. These include both economic and taste/behavioural preference theories. In terms of economic theories, studies link higher cigarette prices with constrains on food expenditure budget which then impacts on children’s nutrition and health [28, 29]. Evidence further indicates that tobacco expenditure crowds out spending on food, with declines in both quantity and quality of food consumed in lower socioeconomic households [30–33].

The taste/behavioural preference theory is based on findings that smokers’ taste sensitivity is potentially altered and suppressed by nicotine and other chemicals found in cigarettes [34, 35] which could lead smokers to consume unhealthy foods [36, 37]. This is supported by research showing that smokers tend to have and an unhealthier diet than non-smokers with a preference for high energy and high fat foods [38–40]. This includes higher intakes of saturated fat and significantly lower intakes of fruits and vegetables [41]. As parents engage in the selection of foods for families, this theory posits that the taste preference for these ‘high flavour’ foods will lead to purchasing of these products for families and higher exposure to unhealthy foods for the children.

Considering that both taste preferences in tobacco users and economic constraints from tobacco use likely impact food choices and availability in households where parents smoke, increased availability of unhealthy food may directly impact caloric intake and risks of childhood obesity. Through analysis of a large longitudinal Australian cohort study of children, this paper aims to investigate the impact of cigarette use on the risk of obesity in children through changes in taste and food choice preferences. Based on the extensive literature to date, it is again hypothesised that parental smoking will be associated with increased obesity in offspring in both childhood and adolescence. It is also hypothesised based on previous research that maternal smoking has additive impacts above that of paternal smoking on childhood obesity. We further explore whether family size and birth order impact potential levels of obesity in offspring of smokers. Finally, based on the food taste preference and economic theories, we hypothesise that parental smoking will be associated with unhealthy food preferences and investigate whether this is impacted by income status.

## Methods

### Participants

The study utilises six waves (2004–2014) of a unique dataset of Australian children from the Longitudinal Study of Australian Children (LSAC) [42–44], a major study following the development of 10,000 children and families across Australia. The LSAC is a nationally representative survey that has been conducted every two years since 2004. Both face-to-face interviews and questionnaires sent out and retrieved via mail are used to collect information on a wide range of topics such as household demographics, health status, education, finance, lifestyle, the relationship history of parents and parenting practices. Participating families were selected at the time of the first survey in 2004 using a two-stage clustered sampling design with postcodes used as the primary sampling unit (PSU). Data on the child and their family’s social circumstances were collected through a face-to face interview (wave 1) and a computer assisted interview (waves 2–6) with the child’s primary carer who in most cases was the mother. More sensitive information was collected from each parent separately using self-completion questionnaires. To ensure a proportional geographic representation of the population, postcodes were selected as a stratified sample by state of residence, and urban and rural geographical status. The sampling frame for the second stage consisted of children born in the selected PSUs. Two age cohorts were selected, infants aged 0–1 year (B cohort) and children aged 4–5 years (K cohort). In this study, we use the K cohort that comprises a sample of approximately 5,000 children. Attrition rates from wave 2 through wave 6 in our dataset are 10.7%, 13.3%, 16.7%, 21.0% and 29.5% relative to wave 1, respectively. The primary estimation sample used in this study consists of an unbalanced panel of participants who have non-missing information on the main outcome variables and covariates.^1^ Table A1 in the Appendix provides summary statistics of variables used in the study.

### Materials

#### Child obesity status

Children’s BMI is calculated from measures of child height and weight that are collected by clinicians using digital scales and a stadiometer during clinical assessment in every wave of the LSAC. Children are then classified as obese (BMI > 30) using cut-off points developed by Cole et al. [45].

#### Parental smoking

Information on parental smoking is derived from the self-completed questionnaires, with detailed information of mothers’ and fathers’ smoking behaviour. In particular, participants are asked the following questions: *Do you currently smoke cigarettes? How often do you currently smoke cigarettes? How many cigarettes do you usually smoke in one day?* Using this information, we construct a dummy variable (smoking status) for parental smoking that equals one if either father or mother is a current smoker. We also construct smoking statuses separately for mothers and fathers. Additionally, we construct two measures of mothers’ and fathers’ smoking to test the robustness of our results: (i) the frequency of smoking (0 – do not smoke, 1 – less than once a day, 2 – at least once a day) and (ii) the number of cigarettes smoked in a day (0 – do not smoke, 1 – less than once a day, 2 – one to five per day, 3 – six to 10 per day, 4–11 to 20 per day; 5 – more than 20 per day).

#### Child nutrition

To explore the role of nutrition as the potential mechanism via which parental smoking affects child obesity, we extract information on children’s dietary intake using their consumption (serves) of food and drinks in the last 24 h. This ranges from nutritious intakes such as fruit, cooked/raw vegetables, salads, water and low-fat milk to high calories food and drinks such as fries, potato chips, doughnuts, soft drinks, cordial and full cream milk. These classifications are performed using the LSAC nutrition data which are coded into either ‘healthy’ or ‘unhealthy’ foods according to the guidelines provided by the Australian Department of Health.^2^ Socioeconomic status can be a confounding factor in the smoking-food choice relationship. In order to rule out the effect of socioeconomic status, we also examine separately using observed data, the average number of serves of food (by type) that children consume across the sub-samples of smoking and non-smoking parents, by splitting the sample by three income groups: low (deciles 1–3), middle (deciles 4–7) and high (deciles 8–10).

#### Other covariates

Given the rich information in the LSAC, we control for a range of child and parents’ characteristics commonly used in previous research [46, 47]. Child characteristics accounted for include basic demographics and early childhood risk factors such as gender, ethnicity, number of siblings, language spoken at home, birth weight, sedentary behaviour (i.e. hours watching TV or on devices), and indices of outdoor and indoor activities.^3^ We account for parental characteristics that can potentially influence the child’s weight such as mother and father’s age, education level, and mother’s age at birth and employment status. Lastly, we consider a set of household characteristics that include household income, the state/territory in which the child is born and whether the child belongs to a single parent household. To account for parents’ mental health that can confound the relationship between child health and parents’ smoking status, we control for parents’ depression scale scores measured using a Kessler 6 (K6) scale of psychological distress. We also include a set of control variables for four dimensions of parenting style (constant, inductive reasoning, warm, hostile)^4^ that could affect the child emotionally and consequently impact on their physical health. Summary statistics on these covariates are provided in Appendix Table A1 for reference.

#### Children’s age

There is a lack of consensus in the literature on whether children of different ages could be grouped together when modelling child obesity because of various reasons such as physiological changes and lifestyle choices in young adolescents [28, 48]. For example, Nonnemaker and Sur [28] use a sample of children aged 2–10 and exclude those older than 10 as they are more likely to smoke and thus experience direct health effects of smoking. In contrast, Meyer [48] focuses only on the range of age between 9 and 12 years given that this is the important development stage of children. Since the LSAC survey has followed the children across the years when they grew up from 4 to 5 years to 15–16, we conduct the analysis across two age groups. Specifically, we split the sample into two time periods such that the children are 4–11 and 12–16 years old, with the latter age group subject to significant changes in body and behaviour due to puberty.^5^

Following Meyer [48], we test for the robustness of our results by excluding from our sample first-born children and those without any older siblings in the households. This allows us to rule out the effect of disproportionate food portion size in single-child families or where the child is the oldest. It is expected that first-born or single child faces a higher risk of being obese. We then replicate the exercise by restricting to families with two children.

### Procedure

**Fig. 1:**
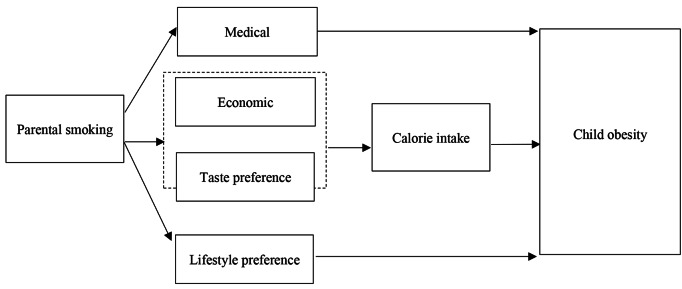
Conceptual model of pathways from parental smoking to child obesity

In this study, we were particularly interested in exploring the channels through which parental smoking impacts body weight in children. Figure 1 identifies a number of pathways from the literature on the effect of parental smoking on child obesity.

In the empirical analysis, we follow a household and health production framework developed by Grossman and Becker [49, 50]. We specify a child health production function in which parental and other inputs are used to produce child health, given an initial health stock. The child health production function is represented as:

1 $${H}_{it}=1(\alpha {+ x}_{it}^{{\prime }}\beta +{\mu }_{i}+{\omega }_{t}+ {\epsilon }_{it}>0)$$

where ${H}_{it}$ is a dichotomous variable for the obesity status of child *i* in year *t* and the indicator function $1$(·) takes the value 1 if the condition in parentheses is valid, 0 otherwise. The vector ${x}_{it}$comprises a set of child, parent and household specific factors, associated with child *i* in year *t*. We also include in ${x}_{it}$ a dummy variable for parents’ smoking status, equal to one if either father or mother is a smoker, and 0 otherwise. ${\mu }_{i}$ represents a vector of unobserved child/parental factors that affect child health. Some of these factors can be time-invariant such as genetic endowment and can be accounted for using a fixed-effects model. However, ${\mu }_{i}$ can also comprise of time varying unobserved factors such as home and neighbourhood environment. If such factors are correlated with parents’ smoking status, then the resulting bias cannot be removed via differencing or fixed-effects estimation. To allow for the possibility of correlation between ${x}_{it}$ and the child-specific effects ${\mu }_{i}$, a correlated random effect model [51] is preferred such that Eq. (1) becomes:

2 $${H}_{it}=1(\alpha +{x}_{it}^{{\prime }}\beta +{\bar{x}}_{it}^{{\prime }}\theta +{\omega }_{t}+ {\epsilon }_{it}>0)$$

where ${\bar{x}}_{i}=1/T{\sum }_{t}{x}_{it}$ are time averages of all time-varying regressors and $t=1,\cdots ,T$. The Mundlak approach effectively separates the individual-specific and time-varying components of unobserved heterogeneity, allowing us to estimate unbiased effects of the ${x}_{it}$’s while controlling for these sources of variation. After estimating the relationship between parents’ smoking behavior and child weight, we next examine the pathways that underly this relationship. As noted above, we aim to find out whether nutritional intake is a mechanism via which parents’ smoking behavior affects their child’s weight. Specifically, we use children’s dietary quality to test the mechanism. We therefore estimate parent’s smoking status as a function of children’s consumption of *healthy* and *unhealthy* food as follows:

3 $${N_{it}} = 1({\delta _0} + x_{it}^\prime \beta + {\epsilon }_{it}>0)$$

where ${N}_{it}$ is dichotomous variable indicating the child consumes a particular type of food. We estimate Eq. (3) for a number of food types using the Mundlak approach. A significant relationship between children’s unhealthy food consumption and parents’ smoking status could be indicative of both, an economic and a taste preference, mechanisms underlying the relationship between parental smoking and child obesity. However, in the absence of expenditure data, we cannot explicitly test the former channel. Irrespective of which channel underlies the relationship between smoking and children’s nutritional intake, the unequivocal conclusion is that any policy that curbs parental smoking will help address child obesity.

### Data availability

The Longitudinal Study of Australian Children (LSAC) is conducted in partnership between the Department of Social Services, the Australian Institute of Family Studies and Roy Morgan. The data is available to approved researchers from government, academic institutions and non-profit organisations. General Release 6 of the LSAC has been used in this study. Access to the LSAC data is available through Dataverse [52].

## Results

### Impact of parental smoking status on childhood obesity

**Fig. 2:**
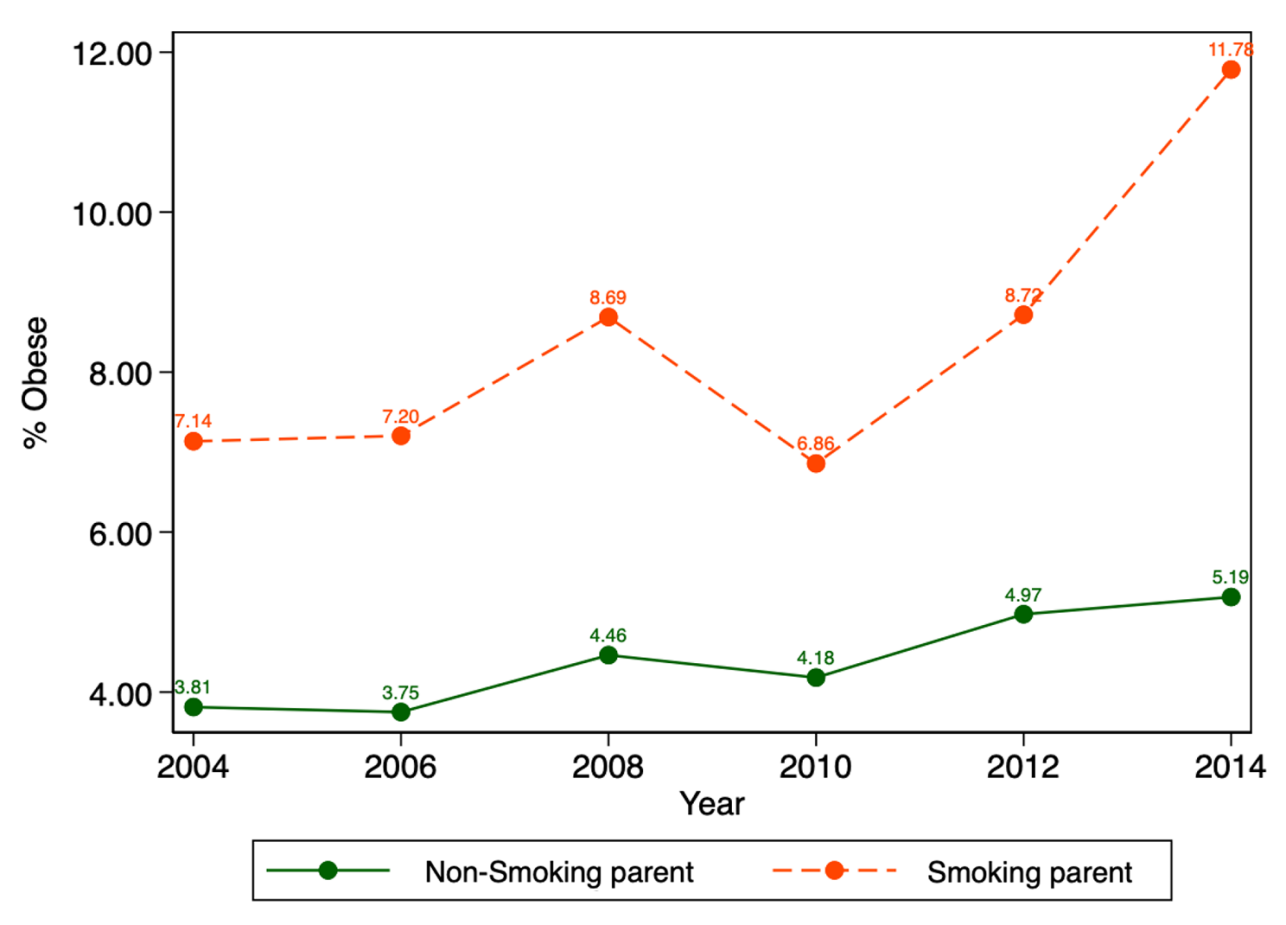
Child obesity by smoking status. *Notes*: Parental smoking status refers to either mother or father reported as a current smoker

Figure 2 presents obesity trends in children across the six waves of the LSAC among parents who currently smoke versus those who do not. The results demonstrate rates of child obesity increasing between 2004 and 2014 in both groups. Clearly, the proportion of obese children is higher in households where parents smoke across all waves of the LSAC.

The link between parental smoking and children’s obesity status is analysed utilising the econometric model (Eqs. 1 and 2) outlined in the procedure section. The results are reported in Table 1. Since the coefficients are hard to interpret (although the significance and direction of the effects are interpretable), we also report marginal effects for ease of interpretation.^6^ From Column 1 we can see a positive and statistically significant relationship between parental smoking status and children’s propensity to be obese (full results available in Appendix Table A2). The marginal effect indicates that children whose parents smoke have a greater risk of being obese. Specifically, the marginal effect of 2.0 means that, holding all other variables constant, children living with parents who smoke have a 0.02 or 2.0% points (pp) higher probability of being obese compared to a child whose parents do not smoke.^7^

**Table 1:** Parental smoking and child obesity – by parental gender

	Both parents	Maternal smoking			Paternal smoking		
	Smoking Dummy	Smoking Dummy	Smoking Frequency	Number of Cigarettes	Smoking Dummy	Smoking Frequency	Number of Cigarettes
	(1)	(2)	(3)	(4)	(5)	(6)	(7)
Parental smoking status	0.461***	0.640***	0.314***	0.160***	0.480***	0.270***	0.158***
	(0.124)	(0.134)	(0.073)	(0.038)	(0.142)	(0.077)	(0.042)
Marginal effects (ME)	[0.020]***	[0.028]***	[0.013]***	[0.007]***	[0.021]***	[0.012]***	[0.007]***
	(0.005)	(0.006)	(0.003)	(0.002)	(0.006)	(0.003)	(0.002)
Observations	10,231	10,421	8,048	8,055	10,486	10,382	10,396
Control variables	Yes	Yes	Yes	Yes	Yes	Yes	Yes
Fixed effects	Yes	Yes	Yes	Yes	Yes	Yes	Yes

*Notes*: Coefficients measure the effect of parental smoking on the propensity of the child being obese and the marginal effect is the effect on the probability of being obese; Standard errors in parentheses; Control variables include child age and gender, home language, migrant, weight at birth, breastfed at six months, mother’s education and father’s education, household income, and number of siblings.*** *p* < 0.01, ** *p* < 0.05, * *p* < 0.1.

### Impacts of maternal versus paternal smoking on childhood obesity

Through the modelling, we estimate the effect of smoking on child obesity separately by parental gender (Table 1, Columns 2–7). Our results indicate that both mother (smoking status: 2.8 pp; frequency of smoking: 1.3 pp; number of cigarettes: 0.7 pp) and father’s smoking behaviours (smoking status: 2.1 pp; frequency of smoking: 1.2 pp; number of cigarettes: 0.7 pp), with all three definitions of smoking, positively and significantly increases the likelihood of their child being obese. However, we find that across all three definitions of smoking, maternal smoking has consistently larger effects on their children’s obesity than paternal smoking.

### Impacts of parental smoking on obesity in childhood and early adolescence groups

We find significant associations of parental smoking with both age groups (4–11 and 12–16 years olds), both effects being statistically significant at the 1% level (Table 2, Columns 1–2). We also note a similar magnitude of the marginal effects (2.3 and 2.5 pp) across both age groups.

**Table 2:** Parental smoking and child obesity, by age groups and family structure

	(1)	(2)	(3)	(4)
	Age 4–11	Age 12–16	Excluding first-born children^1^	Two-children families
Parental smoking status	0.524***	0.658***	0.535***	0.691***
	(0.126)	(0.211)	(0.140)	(0.202)
ME	[0.023]***	[0.025]***	[0.025]***	[0.029]***
	(0.005)	(0.008)	(0.007)	(0.009)
Observations	9,688	4,672	7,871	4,387
Control variables	Yes	Yes	Yes	Yes
Fixed effects	Yes	Yes	Yes	Yes

Notes: Coefficients measure the effect of parental smoking on the propensity of the child being obese and the marginal effect is the effect on the probability of being obese; ^1^also excludes children without any older siblings in the household; Standard errors in parentheses; ME: marginal effects; Control variables are the same as in Table 1; *** *p* < 0.01, ** *p* < 0.05, * *p* < 0.1.

### Impacts of parental smoking based on birth order and family size

Neither birth order nor family size seem to affect our results significantly. [48] Table 2 shows a 0.025 pp higher probability of being obese when we exclude first-born children and those without any older siblings in the households. The results remain consistent when we restrict to families with two children.

### Relationship between smoking status and healthy/unhealthy food choices in their children (by income level)

Based on observed data, our results in Table 3 indicate that children living with parents who are smokers, on average, eat higher number of serves of unhealthy food (such as chips, snacks, and soft drinks) and lower serves of healthy food (such as fruits, cooked vegetables, and water).^8^ Except for the group of food labelled under chocolate, the differences across the two samples are statistically significant across all types of unhealthy food intake at the 5% level of significance. Table 3 also identifies that these results are similar across all economic levels (low, medium and high), highlighting that unhealthy food choices occur across households at all income levels.

**Table 3:** Child food consumption and smoking status – by income groups

***(A) Low-income***		Non-smoking parents	Smoking parents	Mean difference *p*-value^1^
Healthy food	Fresh fruits	1.516	1.452	0.050
	Cooked vegetables	1.092	1.028	0.051
	Skim milk & products	0.241	0.202	0.096
	Water	2.158	1.986	0.000*
	Raw vegetables	0.628	0.543	0.004*
Unhealthy food	Fruit juice	0.824	0.945	0.000*
	Sausage^2^	0.329	0.445	0.000*
	Fries^3^	0.269	0.326	0.009
	Snacks^4^	0.476	0.609	0.000*
	Chocolate^5^	0.930	0.918	0.685
	Full cream milk & products	1.372	1.469	0.004*
	Soft drink & cordial	0.552	0.861	0.000*
Observations		1,871	943	
***(B) Middle-income***		Non-smoking parents	Smoking parents	Mean difference *p*-value^1^
Healthy food	Fresh fruits	1.556	1.478	0.000*
	Cooked vegetables	1.065	0.986	0.000*
	Skim milk & products	0.298	0.231	0.000*
	Water	2.211	2.088	0.000*
	Raw vegetables	0.663	0.534	0.000*
Unhealthy food	Fruit juice	0.836	0.910	0.001*
	Sausage^2^	0.324	0.406	0.000*
	Fries^3^	0.253	0.337	0.000*
	Snacks^4^	0.492	0.622	0.000*
	Chocolate^5^	0.962	0.903	0.003*
	Full cream milk & products	1.381	1.406	0.301
	Soft drink & cordial	0.559	0.721	0.000*
Observations		5,281	1,779	
***(C) High-income***		Non-smoking parents	Smoking parents	Mean difference *p*-value^1^
Healthy food	Fresh fruits	1.656	1.540	0.000*
	Cooked vegetables	1.119	1.063	0.074
	Skim milk & products	0.370	0.340	0.271
	Water	2.374	2.262	0.000*
	Raw vegetables	0.772	0.668	0.001*
Unhealthy food	Fruit juice	0.782	0.888	0.001*
	Sausage^2^	0.317	0.385	0.002*
	Fries^3^	0.230	0.307	0.000*
	Snacks^4^	0.460	0.517	0.018
	Chocolate^5^	0.902	0.934	0.240
	Full cream milk & products	1.339	1.399	0.075
	Soft drink & cordial	0.433	0.610	0.000*
Observations		5,055	852	

*Notes*: ^1^A t-test for the difference of means between the non-smoking and smoking parental groups was conducted. ^2^ Includes meat pie, hamburger, hotdog, sausage and sausage roll. ^3^ Includes hot chips and French fries. ^4^ Includes potato chips, savoury snacks such as Twisties etc. ^5^ Includes biscuits, doughnuts, cake, pie.

Next, we estimate an econometric model (Eq. 3) to account for any unobserved and/or confounding factors that can potentially bias our estimates. Given the dataset spans over a period of time when the children have grown up from 4 to 6 to 14–16 years old it would be inappropriate to conduct the econometric analysis on the basis of number of serves (even after controlling for the age effect). Instead, we use indicators taking value of one if the child consumes a particular food and zero otherwise. We estimate the model separately for each of 12 foods and drinks, regressing it on parental smoking and all the control variables used in the earlier analysis (i.e. Table A2). We also examine these relationships separately with the mother and father’s smoking status. Due to lack of space, we only report the marginal effects of the smoking variable from each equation. They are summarised in Table 4. Across all three specifications (Columns 1–3), we find strong evidence of a positive correlation between parental smoking and their children’s consumption of high calorie unhealthy foods and drinks. Children living with parents who smoke are more likely to consume unhealthy food such as fruit juice, sausage, fries, snacks, full fat milk & products, and soft drinks. Children of parents who smoke are 4.0 pp, 3.4 pp, 6.0 pp and 8.0 pp more likely to consume the groups of foods bundled under the sausage, fries, snacks and soft drinks labels, respectively. We also observe a negative significant relationship between parental smoking and the consumption of skim milk. We conclude that parental smoking affects children’s food intake through a taste preference channel.

**Table 4:** Parental smoking and child nutrition (Marginal Effects)

		(1)	(2)	(3)
		Parental smoking	Mother smoking	Father smoking
Healthy foods	Fresh fruits	0.012	0.016	0.009
		(0.008)	(0.010)	(0.009)
	Cooked	-0.015	-0.010	-0.002
	vegetables	(0.011)	(0.013)	(0.012)
	Skim milk	-0.042***	-0.044***	-0.034***
	& products	(0.010)	(0.013)	(0.011)
	Water	0.003	0.006	0.002
		(0.004)	(0.005)	(0.005)
Unhealthy foods	Raw vegetables	-0.008	-0.006	-0.007
		(0.013)	(0.015)	(0.014)
	Fruit juice	0.017	0.029*	0.002
		(0.013)	(0.016)	(0.014)
	Sausage^1^	0.040***	0.045***	0.032***
		(0.011)	(0.013)	(0.012)
	Fries^2^	0.034***	0.032***	0.019*
		(0.010)	(0.012)	(0.011)
	Snacks^3^	0.060***	0.091***	0.041***
		(0.012)	(0.014)	(0.013)
	Chocolate^4^	-0.036***	-0.030**	-0.031***
		(0.011)	(0.013)	(0.012)
	Full cream milk	0.046***	0.031**	0.050***
	& products	(0.011)	(0.013)	(0.012)
	Soft drink	0.080***	0.084***	0.071***
	& cordial	(0.012)	(0.015)	(0.013)
Observations		10,487	10,487	10,487

*Notes*: Standard errors in parentheses; Estimated using Mundlak model; Results presented as marginal effects; Dependent variable is a dummy which represents whether a child had the above foods or drinks in the last 24 h; Control variables include child age and gender, home language, migrant, weight at birth, breastfed at six months, mother’s education and father’s education, household income, and number of siblings. ^1^ Includes meat pie, hamburger, hotdog, sausage and sausage roll. ^2^ Includes hot chips and French fries. ^3^ Includes potato chips, savoury snacks such as Twisties etc. ^4^ Includes biscuits, doughnuts, cake, pie. *** *p* < 0.01, ** *p* < 0.05, * *p* < 0.1.

## Discussion

This study aims to explore the relationship between parental smoking behaviour and childhood risks of obesity in their children. The results of the study overall demonstrate support for this relationship and highlight the small but significant impact that this modifiable health behaviour may have on children.

The first hypothesis that parental smoking would be associated with increased risks of higher obesity is supported with evidence indicating an increased risk of obesity in children of parents who smoke cigarettes. The impacts of parental smoking are evident with similar magnitude of effects across both age groups. This data confirms a wide range of previous research indicating a clear relationship between parental smoking and childhood obesity (e.g., see analysis of cohort studies by Jaakkola et al. [14]. Whilst the risk of childhood obesity and parental smoking is quite clear, it is important to view adolescent findings with some degree of caution as there is a lack of consensus in the literature on whether or not children of different ages could be grouped together when modelling child obesity because of physiological changes and lifestyle choices in young adolescents [28, 48]. For example, Nonnemaker and Sur [28] use a sample of children aged 2–10 and exclude those older than 10 as they are more likely to smoke and thus experience direct health effects of smoking. In contrast, Meyer [48] focuses only on the range of age between 9 and 12 years given that this is the important development stage of children. We split the sample into two time periods such that the children are 4–11 and 12–16 years old respectively, with the latter age group subject to significant changes in body and behaviour when they reach puberty. This provides insight on both age groups that highlight the increased risk overall of this behaviour from parents.

The hypothesis that maternal smoking would infer greater risks of childhood obesity is also supported with the results indicating that maternal smoking behaviour has a significantly greater negative impact on childhood obesity than paternal smoking behaviour. This finding is in line with the literature on the gender dimension of household food and nutrition security, which indicates the influence of the mother over intrahousehold allocation of resources [53–55]. As the primary caregivers of their children, it is possible that mothers who smoke have a more significant influence on their children’s diet. Notably, this study did not assess whether maternal smoking occurred during pregnancy which a recent meta-analysis has shown is a highly vulnerable stage for increasing risks of childhood obesity due to intra-uterine effects of maternal smoking [56]. This said, other research indicates that even following the perinatal period, the rates of childhood obesity were still greater when fathers or both parents smoked in the household antenatally versus non-smokers [23].

The hypotheses that family size and birth order would impact childhood obesity risk is not supported, with our results highlighting that the relationship between parental smoking and childhood obesity are not an artifact of these factors. This allows us to rule out the effect of disproportionate food portion size in single-child families or where the child is the oldest. Our results show significant effects of parental smoking across both family structures. Even when the analysis restricted the sample to families with two children, there is no evidence for a birth or family size effect on childhood obesity in relation to smoking behaviour.

Given evidence of the relationship between parents’ smoking behaviour and children’s weight, we explore the mechanisms that underlies this relationship. Specifically, we hypothesise that the increased rates of childhood obesity associated with parental smoking (hypotheses 1–3) were associated with childhood nutrition. This included an assessment of the economic and food preference theories as mechanism underlying this relationship between parental smoking and food choices that in turn, impact childhood obesity. The hypothesis that food preferences varied between smoking and non-smoking parents was supported, with clear evidence that children living with parents who are smokers, on average, eat higher number of serves of unhealthy food (such as fries, snacks, and soft drinks) and lower serves of healthy food (such as fruits, cooked vegetables, and water), than children living with non-smokers. This result extends on previous research indicating higher rates of unhealthy food choices in smokers [41] and extended this observation to their children.

A heightened preference for unhealthy food in households where the mother and/or father smoke, could lead to child obesity. The role of family eating habits has been studied extensively in the context of child obesity. For example, Anderson [57] shows that there is a strong negative correlation between maternal employment and days per week having family breakfast/dinner, which possibly explains a higher probability of child obesity. Other studies have shown that children of working mothers spend less time on grocery shopping or cooking or consume a greater share of meals and snacks from away-from-home sources [58–60]. Our study contributes to the literature by reaffirming the important role of nutrition albeit from a taste preference perspective. More broadly, our results indicate that family health behaviours play an important role in children’s health.

### Limitations

Several limitations of the study should be noted. There is a significant body of evidence linking pre- and post-natal exposure to parental smoking with the risk of obesity in childhood and adulthood. According to the developmental origins of health and disease hypothesis (DOHAD), environmental conditions both before and immediately after birth may result in persistent adaptations including alterations in metabolism [21, 61]. On the one hand, children exposed to cigarette smoking in utero and post-natal have a lower birth weight compared to children of non-smokers, while on the other hand, these newborns are at an increased risk of being overweight and obese as children and young adults. Since most of the parents who report themselves as current smokers in our sample also smoked when the mother was pregnant with the child, obesity in childhood or early adolescence can be associated with pre- and post-natal exposure to smoking. There are a number of potential confounders that can also influence child obesity, such as parents’ BMI, genetics, children’s sleep patterns and amount of screen time. Unfortunately, we do not have such information in the dataset. Finally, as with any self-reported data, consumption of tobacco may be under-reported due to social stigma associated with smoking.

## Conclusion

This paper provides empirical evidence of the association between parental smoking and childhood obesity using a unique dataset of Australian children aged 4–16 years, addressing for the potential endogeneity of parental smoking. It contributes to the existing evidence linking parental smoking to childhood obesity, showing that children of parents who smoke are at a noticeable risk of developing obesity compared to children of non-smoking parents, regardless of income level, children age, family size and birth order. Although maternal smoking seems to have more impact on children obesity, parental smoking overall is significantly linked to unhealthy food choices. While further research is needed to elucidate the exact mechanisms underlying this association, these findings underscore the importance of tobacco control efforts and targeted interventions to reduce parental smoking and protect children. Finally, our findings underscore the need for tobacco control measures that help parents quit smoking or reduce their tobacco use as they can have positive spillover effects on family health behaviours, including dietary choices and physical activity. They also highlight the importance of strategies that promote healthy family behaviours including dietary choices and physical activity, all of which can be beneficial for child health and obesity prevention.

### Electronic supplementary material

Below is the link to the electronic supplementary material.

Supplementary Material 1

## Data Availability

The data is available to approved researchers from government, academic institutions and non-profit organisations. General Release 6 of the LSAC has been used in this study. Access to the LSAC data is available through Dataverse.

## Footnotes

1 To test for missing variable bias in our study, we employ the inverse probability weighting (IPW) suggested by Fitzgerald et al. (1998). Weights are derived as the inverse of the estimated propensity of the ones that remain in the sample during the entire period and are incorporated into our estimation analysis. The results are qualitatively similar to those estimated without weights which indicates that there are no selection concerns and that the missing observations are most likely random.

2
See Get Up & Grow – Healthy eating and physical activity for early childhood – Resource collection | Australian Government Department of Health and Aged Care.

3 This includes activities such as cooking, caring for pet, and drawing.

4 Refer to https://growingupinaustralia.gov.au/sites/default/files/tp12.pdf for more information on measures of parenting styles in the LSAC.

5 Note that according to the latest National Drug Strategy Household Survey (Australian Institute of Health and Welfare, 2017), about 1.6% of children aged 12–17 reported smoking daily in 2016.

6 In such a binary probit model, we essentially estimate a latent model where the dependent variable is the propensity of the child being obese. The marginal effect measures the effect of a given X on the probability of being obese.

7 For a continuous variable, the marginal effect represents the change in the probability of being obese, when the X variable increases by one unit, holding all other variables constant.

8 Note that the rate of prevalence of smoking in the three income groups is 34%, 25% and 15% respectively.
