# Supplementary material for: The links between parental smoking and childhood obesity: data of the longitudinal study of Australian children
Source: BMC Public Health. 2024 Jan 2;24:68. doi: 10.1186/s12889-023-17399-5 (PMC10762820; doi:10.1186/s12889-023-17399-5)
Supplement: Supplementary file 1 — Supplementary Material 1 [file 12889_2023_17399_MOESM1_ESM.docx]

**Appendix A**

**Table A1: Summary statistics**

|  | Observ. | Mean | St. Dev | Min | Max |
| --- | --- | --- | --- | --- | --- |
| ***Child weight status*** |  |  |  |  |  |
| Obesity (dummy) | 24,743 | 0.06 | 0.24 | 0 | 1 |
| ***Parental smoking*** |  |  |  |  |  |
| Parental smoking status (dummy) | 15,781 | 0.23 | 0.42 | 0 | 1 |
| Mother’s smoking status (dummy) | 22,853 | 0.18 | 0.39 | 0 | 1 |
| Father’s smoking status (dummy) | 16,381 | 0.18 | 0.38 | 0 | 1 |
| *Mother’s smoking (frequency)* |  |  |  |  |  |
| Does not smoke | 22,726 | 0.82 | 0.39 | 0 | 1 |
| Less than once a day | 22,726 | 0.02 | 0.14 | 0 | 1 |
| At least once a day | 22,726 | 0.16 | 0.37 | 0 | 1 |
| *Father’s smoking (frequency)* |  |  |  |  |  |
| Does not smoke | 13,605 | 0.82 | 0.38 | 0 | 1 |
| Less than once a day | 13,605 | 0.02 | 0.12 | 0 | 1 |
| At least once a day | 13,605 | 0.16 | 0.37 | 0 | 1 |
| *Mother’s smoking (number of cigarettes)* |  |  |  |  |  |
| Does not smoke | 22,618 | 0.82 | 0.38 | 0 | 1 |
| Less than one a day | 22,618 | 0.01 | 0.12 | 0 | 1 |
| 1 to 5 per day | 22,618 | 0.03 | 0.17 | 0 | 1 |
| 6 to 10 per day | 22,618 | 0.06 | 0.23 | 0 | 1 |
| 11 to 20 per day | 22,618 | 0.06 | 0.24 | 0 | 1 |
| More than 20 per day | 22,618 | 0.02 | 0.13 | 0 | 1 |
| *Father’s smoking (number of cigarettes)* |  |  |  |  |  |
| Does not smoke | 13,561 | 0.83 | 0.38 | 0 | 1 |
| Less than one a day | 13,561 | 0.01 | 0.11 | 0 | 1 |
| 1 to 5 per day | 13,561 | 0.02 | 0.15 | 0 | 1 |
| 6 to 10 per day | 13,561 | 0.04 | 0.19 | 0 | 1 |
| 11 to 20 per day | 13,561 | 0.07 | 0.25 | 0 | 1 |
| More than 20 per day | 13,561 | 0.03 | 0.18 | 0 | 1 |
| ***Parental and child characteristics*** |  |  |  |  |  |
| Child age (in years) | 25,440 | 9.48 | 3.47 | 4.25 | 15.83 |
| Gender (female = 1) | 25,440 | 0.49 | 0.50 | 0 | 1 |
| Home language (English=1) | 25,435 | 0.89 | 0.31 | 0 | 1 |
| Migrant (Yes=1) | 25,440 | 0.04 | 0.20 | 0 | 1 |
| Weight at birth | 25,097 | 3.41 | 0.59 | 0.54 | 6.13 |
| Breastfed at 6 months (Yes=1) | 25,323 | 0.59 | 0.49 | 0 | 1 |
| Single mom (Yes=1) | 25,419 | 0.21 | 0.41 | 0 | 1 |
| *Mother education* |  |  |  |  |  |
| Mother – University | 21,721 | 0.31 | 0.46 | 0 | 1 |
| Mother – Advance | 21,721 | 0.47 | 0.50 | 0 | 1 |
| Mother – Year 12 | 21,721 | 0.08 | 0.28 | 0 | 1 |
| Mother – Less than year 12 | 21,721 | 0.14 | 0.34 | 0 | 1 |
| *Father education* |  |  |  |  |  |
| Father – University | 25,440 | 0.31 | 0.46 | 0 | 1 |
| Father – Advance | 25,440 | 0.40 | 0.49 | 0 | 1 |
| Father – Year 12 | 25,440 | 0.12 | 0.32 | 0 | 1 |
| Father – Less than year 12 | 25,440 | 0.16 | 0.37 | 0 | 1 |
| Number of siblings | 25,419 | 1.57 | 1.06 | 0 | 11 |
| Household income (in log) | 25,319 | 7.38 | 0.69 | 0.34 | 11.31 |
| Mother chronic problem (Yes=1) | 25,007 | 0.03 | 0.16 | 0 | 1 |
| Father chronic problem (Yes=1) | 21,712 | 0.03 | 0.16 | 0 | 1 |
| Mother depression scale | 23,198 | 4.43 | 0.62 | 1 | 5 |
| Father depression scale | 16,435 | 4.51 | 0.55 | 1 | 5 |
| Hours watching TV in weekday | 21,834 | 2.95 | 0.77 | 1 | 5 |
| Hours watching TV in weekend | 21,827 | 3.34 | 0.89 | 1 | 5 |
| Outdoor activities index | 25,300 | 2.55 | 1.26 | 0 | 5 |
| Home activities index | 17,934 | 1.39 | 0.62 | 0 | 3 |
| Mother – consistent parenting | 23,960 | 4.13 | 0.65 | 1 | 5 |
| Father – consistent parenting | 16,571 | 4.05 | 0.66 | 1 | 5.25 |
| Mother – hostile parenting | 23,965 | 2.15 | 0.63 | 1 | 5 |
| Father – hostile parenting | 16,570 | 2.16 | 0.63 | 1 | 5 |
| Mother – inductive parenting | 23,961 | 4.13 | 0.74 | 1 | 5 |
| Father – inductive parenting | 16,579 | 3.87 | 0.76 | 1 | 5 |
| Mother – warm parenting | 23,972 | 4.30 | 0.58 | 1 | 5 |
| Father – warm parenting | 16,591 | 3.95 | 0.67 | 1 | 5 |
| *Notes:* Parental smoking is identified as either mother or father is a smoker; Reference group for parental education is ‘not completed year 12’; higher depression scale represents higher levels of non-specific psychological distress; higher outdoor/home activities index indicates more frequent activities. Higher parenting scores are associated with better parenting style but hostile parenting. | | | | | |

**Table A2: Parental smoking and child obesity – Full results**

| Variables | Coeff | Variables | Coeff |
| --- | --- | --- | --- |
|  | (SE) |  | (SE) |
| Parental smoking status | 0.461*** | (continued) |  |
|  | (0.124) |  |  |
| Gender (female = 1) | 0.208 | Father depression scale | 0.126 |
|  | (0.130) |  | (0.093) |
| Home language (English=1) | -0.809*** | Hours watching TV in weekday | 0.186** |
|  | (0.198) |  | (0.073) |
| Migrant | 0.101 | Hours watching TV in weekend | 0.106* |
|  | (0.327) |  | (0.060) |
| Weight at birth | 0.692*** | Outdoor activities index | -0.006 |
|  | (0.127) |  | (0.041) |
| Breastfeed in six months | -0.405*** | Home activities index | -0.014 |
|  | (0.134) |  | (0.084) |
| Number of siblings | -0.051 | Mother – consistent parenting | -0.217** |
|  | (0.062) |  | (0.089) |
| Household income (in log) | -0.065 | Father – consistent parenting | -0.211** |
|  | (0.098) |  | (0.082) |
| Mother – University | -0.385* | Mother – hostile parenting | -0.062 |
|  | (0.219) |  | (0.096) |
| Mother – Advance | 0.065 | Father – hostile parenting | 0.052 |
|  | (0.173) |  | (0.094) |
| Mother – Year 12 | -0.434 | Mother – inductive parenting | 0.002 |
|  | (0.276) |  | (0.081) |
| Father – University | -0.042 | Father – inductive parenting | 0.017 |
|  | (0.203) |  | (0.075) |
| Father – Advance | -0.125 | Mother – warm parenting | 0.115 |
|  | (0.174) |  | (0.119) |
| Father – Year 12 | -0.292 | Father – warm parenting | -0.046 |
|  | (0.221) |  | (0.099) |
| Mother chronic problem | 0.157 | Single parent family | 0.017 |
|  | (0.299) |  | (0.197) |
| Father chronic problem | -0.115 | Mother age at birth | -0.018* |
|  | (0.287) |  | (0.011) |
| Mother depression scale | -0.041 | Mother is unemployed | 0.079 |
|  | (0.090) |  | (0.117) |
| Fixed effects | Yes | | |
| Observations | 10,231 | | |

*Notes:* Coefficients measure the effect of parental smoking on the propensity of the child being obese; Standard errors in parentheses; *** p<0.01, ** p<0.05, * p<0.1.
